# Gas7 Is Required for Mesenchymal Stem Cell-Derived Bone Development

**DOI:** 10.1155/2013/137010

**Published:** 2013-06-06

**Authors:** Chuck C.-K. Chao, Feng-Chun Hung, Jack J. Chao

**Affiliations:** Department of Biochemistry and Molecular Biology and Institute of Biomedical Sciences, College of Medicine, Chang Gung University, Taoyuan 333, Taiwan

## Abstract

Mesenchymal stem cells (MSCs) can differentiate into osteoblasts and lead to bone formation in the body. Osteoblast differentiation and bone development are regulated by a network of molecular signals and transcription factors induced by several proteins, including BMP2, osterix, and Runx2. We recently observed that the growth-arrest-specific 7 gene (Gas7) is upregulated during differentiation of human MSCs into osteoblasts. Downregulation of Gas7 using short-hairpin RNA decreased the expression of Runx2, a master regulator of osteogenesis, and its target genes (alkaline phosphatase, type I collagen, osteocalcin, and osteopontin). In addition, knockdown of Gas7 decreased the mineralization of dexamethasone-treated MSCs in culture. Conversely, ectopic expression of Gas7 induced Runx2-dependent transcriptional activity and gene expression leading to osteoblast differentiation and matrix mineralization. Genetic mutations of the Gas7 gene increased body fat levels and decreased bone density in mice. These results showed that Gas7 plays a role in regulating the pathways which are essential for osteoblast differentiation and bone development. In this review, we summarize the involvement of Gas7 in MSC-based osteogenesis and osteoporosis and describe the possible mechanisms responsible for the maintenance of cellular homeostasis in MSCs and osteoblasts.

## 1. Gas7: A *Pombe* Cdc15 Homology Protein

The Gas7 protein is part of the Pombe Cdc 15 homology (PCH) family which belongs to the proline, serine, threonine-rich phosphatase interacting protein (PSTPIP) subfamily [1, 2]. Gas7 was initially identified as an upregulated gene in NIH3T3 cells cultured without serum, and the structure of the encoded protein showed homology to Oct2 and synapsins, proteins involved, respectively, in neuron development, and neurotransmitter release [3, 4]. Gas7 is selectively expressed in mature cerebellar neurons, cerebral cortical neurons, and hippocampal neurons [4, 5]. The human Gas7 gene is located on chromosome 17p12 (based on information provided by Ensembl and UDB/GeneLoc). Open reading frame analysis of the 412 amino acid-coding Gas7 gene predicted the production of a 47,266-Da protein. Gas7a and Gas7b protein isoforms, which are obtained by alternative splicing, have also been described [6].

**Figure 1:**
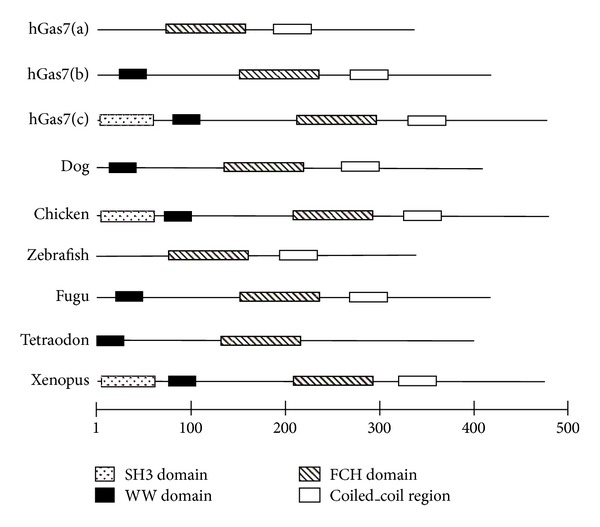
Domain structure of Gas7 protein isoforms. The Gas7 isoform b found in mammals possesses WW, Fes/CIP4 homology (FCH), and coiled-coil domains the Gas7 isoform c possesses an additional SH3 domain at the N-terminus. The number of amino acids for the proteins is indicated.

Several studies have been performed to examine the physiological functions of Gas7 in humans and rodents [3, 7]. These studies have shown that Gas7 is mainly expressed in the brain and is involved in morphological differentiation and neuritogenesis [3, 5–7]. These observations are consistent with the observed Gas7 expression pattern in normal human tissues based on the quantification of expressed sequence tags (ESTs) from various tissues in Unigene clusters. Gas7 isoforms also appear to be differentially expressed and regulated in the brain of rats after hippocampal neuron injury [5]. Recently, the neurite outgrowth of hippocampal neurons was shown to require the binding of Gas7 to N-WASP [8]. This binding required WW-Pro domains—unique to the PCH protein family—and was largely of the SH3-Pro type. These observations indicate that the binding between Gas7 and N-WASP may lead to formation of membrane protrusions, possibly via recruitment of the Arp2/3 complex and independently of Cdc42 [8]. Controlled expression of Gas7 also appears to be critical for tissue development since MLL-GAS7 translocations were detected in individuals suffering of treatment-related acute myeloid leukemia [9]. Other authors showed that Gas7b binds to the WW domain of Tau and that the Gas7b/Tau complex binds to microtubules in Neuro2A cells, a process which promotes tubulin polymerization [10]. Gas7b downregulation was shown to protect neuroblast cells against apoptosis in vitro [11]. Similar Gas7 genes have been identified in other organisms. Comparison of the predicted Gas7 proteins in these various organisms confirmed the conservation of unique protein domains (Figure 1).

These results illustrate that Gas7 is implicated in several cellular processes that are evolutionally conserved in various species. Earlier, we also found a functional link between the expression of Gas7 and the processes of chondrogenesis and osteogenesis in human bone marrow-derived human MSCs [12, 13].

## 2. Mesenchymal Stem Cells

MSCs represent nonhematopoietic stem cells with the capacity to differentiate into various lineages, including osteoblastic, chondrogenic, and adipogenic lineages. Recent studies have shown that MSCs may also differentiate into other lineages, including neuronal and cardiomyogenic ones. Extracellular stimuli enable efficient initiation of mechanotransductive signaling which regulate stem cell fate. Examples include the effects of stereotopography and matrix stiffness on the fate of MSCs [14, 15]. Following their initial detection and isolation from bone marrow, MSCs have been harvested from many other tissues, including adipose tissue, muscles, tendons, placenta, liver, cartilage, spleen, and thymus. Our group has previously demonstrated that density gradient media is an efficient method to isolate marrow-derived human MSCs with osteogenic potential [16]. Their easy isolation and ex vivo expansion along with their immune-privileged nature make MSCs popular candidates for stem cell-based regenerative therapies [17]. MSCs can alter disease pathophysiology in various ways, including by differentiating into various lineages, by leading to cytokine secretion and immune modulation, and by interacting with damaged and diseased tissues. The main characteristics of MSC biology, such as culture, differentiation capabilities, and homing mechanisms, have been extensively reviewed [18]. MSCs found in bone marrow and adipose tissue represent the common precursor which can differentiate into osteoblasts and adipocytes. Several transcription factors and extracellular and intracellular signals regulating adipogenesis and osteoblastogenesis have been identified. For instance, the Wnt/*β*-catenin pathway was shown to induce osteoblastogenesis and to inhibit adipogenesis, whereas the peroxisome proliferator activated receptor-*γ* (PPAR-*γ*) is a potent inducer of adipogenesis and inhibitor of osteoblastogenesis [19].

## 3. Gas7 in MSC-Based Osteogenesis

Over the last two decades, many factors have been identified that regulate cell differentiation. Runx2/Cbfa1 [20, 21], osterix [22], Msh homeobox 2 [23], BMP2 [24], Wnt, and Hedgehog [25] have all been shown to play a role in osteoblastogenesis [26, 27]. Similarly, PPAR-*γ*, CCAAT/enhancer binding protein (C/EBP) *α* and *β*, glucocorticoid receptor (GR), insulin, and Kruppel-like factor 5 (KLF5) have been identified as critical regulators of adipogenesis [28]. Notably, the primary inducer of adipogenesis, PPAR-*γ*, may inhibit osteoblastogenesis [29]. Functional crosstalk between Wnt and PPAR-*γ* signaling pathways regulating MSC differentiation have been discussed in detail [19]. Bone is a special tissue where calcium, osteocalcin, and amino acids represent extracellular signals regulating mineralization. Runx2, for example, represents an essential transcription factor controlling osteoblast differentiation as seen from the observation that Runx2-deficient mice do not form bones due to the lack of osteoblasts [21, 30]. Consistent with these results, Runx2 mutations have been found in patients with cleidocranial dysplasia, a disease characterized by deformities in the collarbone and skull [31]. Following the commitment to osteogenesis mediated by Runx2, development of the bone cell phenotype is reinforced by osterix; accordingly, osterix-deficient preosteoblasts only express chondrocyte gene markers [22]. In addition, Runx2-overepressing mice produce abnormal osteoblasts showing impaired matrix production and mineralization [32, 33]. Therefore, Runx2 plays a critical role in controlling the commitment of multipotent mesenchymal cells to the osteoblastic lineage during bone formation. Several in vitro studies have shown that Runx2 can upregulate bone matrix gene expression, including bone sialoprotein, fibronectin, osteocalcin, osteopontin, and type I collagen [20, 27]. The role of Runx2-dependent transcriptional activation has been demonstrated through analysis of osteocalcin and type I collagen gene promoters [34, 35].

**Figure 2:**
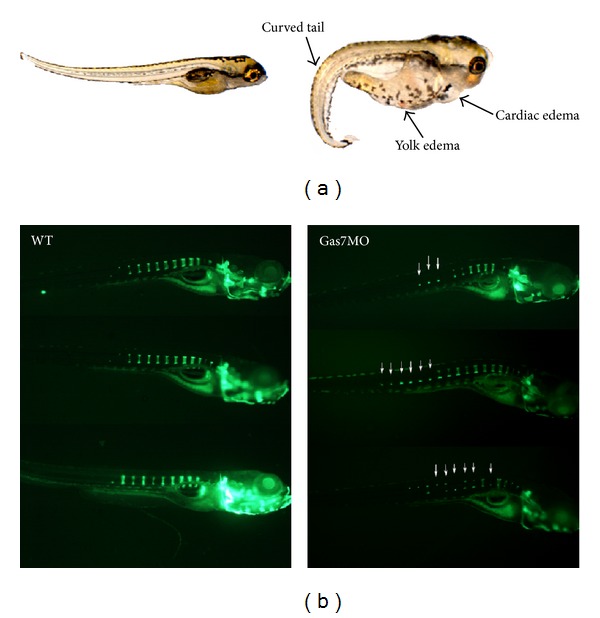
Abnormal bone structure and phenotypes in zebrafish embryos following Gas7 knockdown (Gas7MO). (a) Representative embryos with Gas7MO or control treatment (WT). (b) Abnormal bone structure in embryos with Gas7MO as revealed by fluorescence-labeled bone component.

Recently, we observed that Gas7 represents a novel regulator of MSC-based osteogenesis [13]. Gas7 controls the differentiation of human MSCs into functional osteoblasts by enhancing Runx2-dependent gene expression. We observed that Gas7, specifically the b isoform (Gas7b), was upregulated during dexamethasone-induced differentiation of MSCs into osteoblasts. Downregulation of Gas7 using shRNA reduced the expression of Runx2 and its target genes alkaline phosphatase, osteocalcin, osteopontin, and type I collagen. In addition, knockdown of Gas7 reduced matrix mineralization of dexamethasone-treated MSCs in vitro. In contrast, overexpression of Gas7 induced gene expression associated with osteoblast differentiation and matrix mineralization, and also induced the mineralization of MSCs in vitro. Moreover, by using a gene reporter assay to monitor osteocalcin expression in human MSCs, we showed that Runx2-dependent transcriptional activity was enhanced by ectopic expression of Gas7. These observations demonstrated that Gas7 enhances Runx2-dependent gene expression and represents an endogenous regulator of osteogenic differentiation in human MSCs. The importance of Gas7 in early development was also observed in zebrafish [36]. Gas7 knockdown with Morpholino (MO) antisense oligonucleotides reduced bone density, shortened the vertebral column, and produced a curved body shape in laboratory animals (Figure 2). Gas7 knockdown caused defects that could be rescued by overexpression of Gas7 of either zebrafish or human origin. Further studies are under way to determine whether the regulatory effects of Gas7 require other protein partners. 

## 4. Gas7 in Osteoporosis

**Figure 3:**
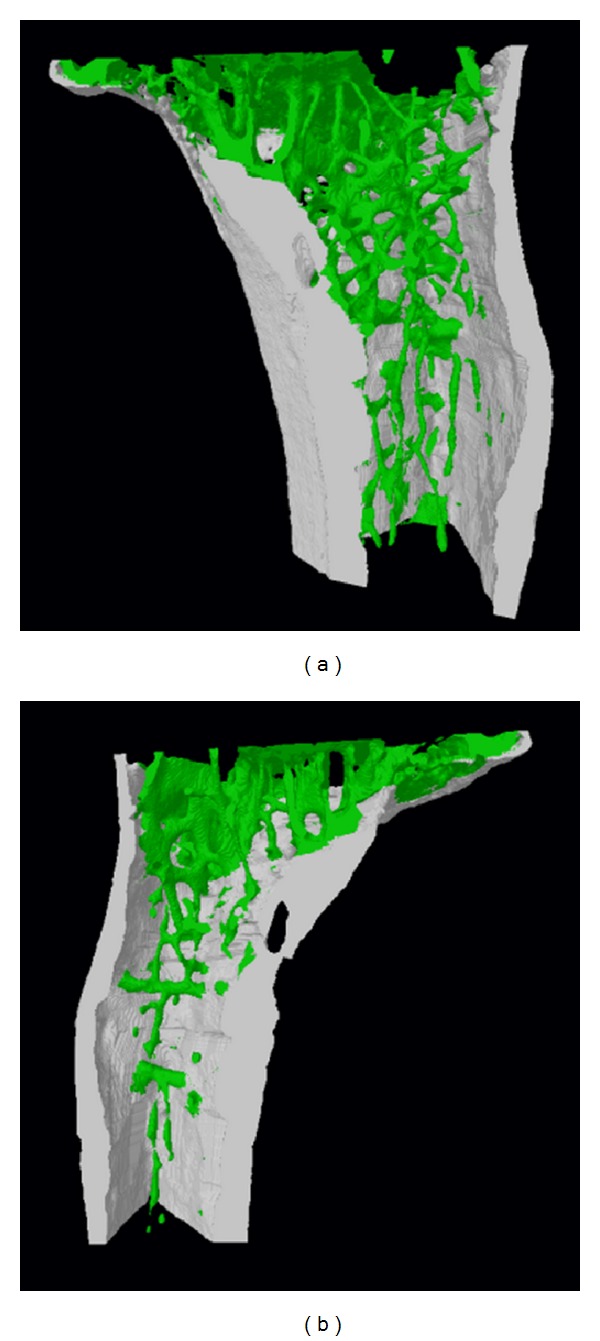
Reduced tibia bone density in gas7-knockout mouse. Volumetric images of trabecular and cortical bone were reconstructed using a commercial surface rendering program from series of micro-CT images. The images support the mass reduction in trabecular bone in gas7-knockout mice (b) compared to the wild-type group (a).

In a recent study, we provided a detailed account of the creation and characterization of Gas7-deficient mice that express a labile Gas7 mutant protein with the same properties of wild-type Gas7 [37]. Our data showed that Gas7 is involved in motor neuron function associated with muscle strength maintenance. Gas7 protein expression was not detected in bone-marrow MSCs prepared from Gas7 mutant mice. Notably, these mutant MSCs showed impaired osteogenesis (unpublished observations). Bone density in wild-type and Gas7 mutant mice was determined (Figure 3). Volumetric images of trabecular and cortical bone for each group were reconstructed using a commercial surface rendering program from series of micro-X-ray computed topography (CT) images. Other calculations of trabecular bone mass and fractal dimension in tibia bones also supported the observations of mass reduction in trabecular bones in male Gas7 mutant mice compared to the wild-type group. Consistent with our findings, other authors have reported that perturbation of Gas7, Me1, or Gpx3 leads to significant changes in mouse obesity-related traits [38]. Among the newly validated genes, Gas7 is thought to be involved in fat metabolism and other pathways, such as the insulin signaling pathway. Given that PPAR-*γ*, C/EBP *α* and *β*, GR, insulin, and KLF5 have been identified as essential regulators of adipogenesis [28], whether and how Gas7 functionally interacts with these transcription factors or their downstream gene products will be an interesting topic in future studies.

## 5. Concluding Remarks

Two signaling pathways that determine the differentiation of MSCs into adipocytes or osteoblasts by suppressing the transactivation function of PPAR-*γ* have been described. These signaling cascades promote osteoblastic differentiation from MSCs via two distinct modes of PPAR-*γ* transrepression. Recent studies indicate that certain differentiation factors may affect the biological activity of other regulators. For instance, differentiation factors regulating osteoblastogenesis inhibit adipogenesis and vice versa. Runx2-dependent gene expression and the differentiation of MSCs into osteoblasts enhance bone formation. Gas7 also plays a role in regulating the pathways, which are essential for osteoblast differentiation and bone development, probably through the induction of undetermined factors that interact with Runx2. The role of Gas7 in regulating these pathways is under intense investigation and may lead to novel therapies. Current therapies for osteoporosis are mainly based on the prevention of bone resorption by treatment with bisphosphonates and selective estrogen receptor modulators [39]. However, serious side effects have been reported for these drugs, particularly an increased incidence of breast and ovarian cancers [40]. Induction of osteoblastogenesis may prove to be beneficial for the treatment of osteoporosis. We expect that shifting the focus from inhibition of bone resorption to stimulation of bone formation may lead to the development of better strategies to prevent and treat osteoporosis.
